# A Retrospective Cohort Study of Red Cell Alloimmunisation in Rural, Remote, and Aboriginal and Torres Strait Islander Peoples Admitted to Intensive Care in the Northern Territory, Australia

**DOI:** 10.3390/jcm12041606

**Published:** 2023-02-17

**Authors:** Tina Noutsos, Maree A. Perry, Paul J. Secombe, David J. Roxby, Romi Sinha, Lewis T. Campbell

**Affiliations:** 1 Menzies School of Health Research, Charles Darwin University, Darwin, NT 0810, Australia; 2 Department of Haematology, Royal Darwin Hospital, Darwin, NT 0810, Australia; 3 College of Medicine and Public Health, Flinders University, Adelaide, SA 5042, Australia; 4 School of Public Health and Social Work, Queensland University of Technology, Brisbane, QLD 4000, Australia; 5 Melbourne Medical School, University of Melbourne, Melbourne, VIC 3010, Australia; 6 Intensive Care Department, Alice Springs Hospital, Alice Springs, NT 0870, Australia; 7 Public Health and Preventive Medicine, Monash University, Melbourne, VIC 3004, Australia; 8 Intensive Care Unit, Royal Darwin Hospital, Darwin, NT 0810, Australia

**Keywords:** transfusion medicine, blood transfusion, erythrocyte transfusion, immunohematology, alloantibodies, Aboriginal health, Aboriginal Australians

## Abstract

Red cell (RC) alloantibodies occur on exposure to non-self RC antigens in transfusion and pregnancy (typically IgG and clinically significant) or in association with non-RC immune environmental factors (typically IgM and not clinically significant). In Australia, the risk of RC alloimmunisation in First Nations peoples is unknown. We assessed the epidemiology, specificity, and antecedents of RC alloimmunisation via a data linkage retrospective cohort study of Northern Territory (NT) intensive care unit (ICU) patients (2015–2019). Of 4183 total patients, 50.9% were First Nations. In First Nations versus non-First Nations patients, the period prevalence of alloimmunisation was 10.9% versus 2.3%, with 390 versus 72 prevalent alloantibodies detected in 232 versus 48 alloimmunised patients, of which 135 (34.6%) versus 52 (72.2%) were clinically significant specificities. Baseline and follow-up alloantibody testing were available for 1367 patients, in whom new incident clinically significant alloantibodies developed in 4.5% First Nations versus 1.1% non-First Nations patients. On Cox proportional hazards modelling, adjusted hazard ratios (HR) showed First Nations status (HR 2.67 (95% CI 1.05–6.80), *p* = 0.04) and cumulative RC unit transfusion exposure (HR 1.03 (95% CI 1.01–1.05), *p* = 0.01) were independent predictors of clinically significant alloimmunisation. First Nations Australian patients are at increased risk of alloimmunisation due to RC transfusion, underscoring the importance of very judicious use of RC transfusions and shared decision-making with patients. Further studies are recommended to explore the role of other (non-RC) immune host factors, given the relative high prevalence of non-clinically significant IgM alloantibodies within alloimmunised First Nations patients.

## 1. Introduction

In certain settings, red blood cell (RC) transfusions can benefit patients; however, they can also cause harm. RC transfusion can assist in correcting anaemia to improve oxygen delivery to the tissues, offsetting risk of organ dysfunction or failure. In emergency settings, such as life-threatening haemorrhage, RC transfusion can save lives [1]. However, transfusion also carries some risk of harm. RC transfusion is an independent predictor for death and increased hospital length of stay after adjustment for anaemia and major haemorrhage [1,2]. RC transfusion is also associated with adverse events, including haemolytic transfusion reactions [3,4].

Both patients and prescribers of blood transfusions may be unaware of these relative benefits versus harms. Amongst transfusion prescribers, there is significant variation in practice with respect to haemoglobin triggers for RC transfusion [5]. Surveys of medical residents have shown deficiencies in transfusion-related patient consent, with prescribers often overstating benefits for which there is poor evidence and inadequately discussing risks [6,7]. Minimum elements of informed consent for RC transfusion include appropriate description of risks, benefits, and treatment alternatives, with a shared decision-making approach.

One adverse consequence of RC transfusion is alloimmunisation, the risks and consequences of which are higher in certain demographic groups. Recipients of RC transfusions may develop alloantibodies to foreign blood group antigens present in the blood donor but not the recipient and during pregnancy on exposure to fetal non-self RC antigens (usually IgG type and clinically significant) or in association with non-RC immune environmental factors, such as bacterial exposure (typically IgM type and usually not clinically significant) [8]. Whilst the ABO and Rhesus D (RhD) blood groups are well known to prescribers of RC transfusions, there are 43 different blood group systems described, including 345 different antigens [9]. RC blood group antigens are genetically determined and differ among demographic groups globally [10]. RC alloantibodies which are clinically significant carry a risk of acute or delayed immune-mediated haemolytic transfusion reactions on exposure to the corresponding RC antigen, and/or pose a risk to future pregnancies in women due to haemolytic disease of the fetus/newborn. The presence of clinically or non-clinically significant RC alloantibodies may also cause delays in finding crossmatch compatible blood in a timely manner for future RC transfusions [8].

In Australia, it is unknown whether Aboriginal and Torres Strait Islander peoples are at higher risk of RC alloimmunisation [2]. Aboriginal and Torres Strait Islander peoples are the Indigenous peoples of Australia and herein respectfully referred to as First Nations peoples. In the context of unmet healthcare needs, anaemia, trauma, and major haemorrhage are more common in First Nations peoples and are major reasons for RC transfusion use [2,11,12]. First Nations peoples of the Northern Territory (NT) of Australia are a culturally and linguistically rich and diverse peoples, predominantly living in remote Australia, speaking over 100 different languages across the NT, and possessing genetic diversity paralleling linguistic diversity [13]. For many First Nations Australian peoples, blood is a sacred substance, with many intertwined and complex cultural and spiritual meanings and connections to family, land, and community [14]. Small studies have shown that the distribution of RC ABO, and RhD blood groups differ among First Nations peoples across northern and central Australia, compared with pooled other Australians [15,16,17]. In the context of Australia’s history of colonisation and the resultant Australian blood donor pool being predominantly Caucasian, resultant RC antigen donor–recipient disparities may confer a higher risk of alloimmunisation for First Nations Australians [15]. Compounding this, there is no existing evidence on RC alloimmunisation for First Nations Australians to inform our understanding of this risk, nor assist in RC transfusion informed consent and shared decision-making approaches.

We investigated the epidemiology and associated predictors of RC alloimmunisation in a five-year cohort of critically ill patients admitted to intensive care units (ICU) in the NT, which includes a high proportion of First Nations peoples, and a case mix of patients with a typically high prevalence of anaemia, bleeding, and RC transfusion use [18]. We hypothesised that exposure to RC transfusion and First Nations status would be positively associated with risk of RC alloimmunisation.

## 2. Materials and Methods

### 2.1. Study Design, Setting, and Participants

We conducted a retrospective data linkage cohort study using existing administrative, laboratory, and clinical datasets covering all relevant demographic, laboratory, and clinical data from all inpatients of NT ICUs over a five-year period.

The NT spans 1.35 million km^2^, with a population of approximately 250,000 people, over 30% of whom identify as Aboriginal and/or Torres Strait Islander (First Nations), with nearly 50% of the NT population residing in remote or very remote Australia [19]. ICU services for the NT are provided by two units with a combined catchment area exceeding 2.5 million km^2^: Royal Darwin Hospital (360-bed hospital), servicing the Top End of the NT and eastern Kimberly region of Western Australia (WA); and Alice Springs Hospital (186-bed hospital), which admits patients from the southern half of the NT, central eastern WA, northern South Australia, and parts of far western Queensland. Together, they provide specialist intensive care to approximately 1800 patients per year.

Patients eligible for inclusion in this study were those admitted to NT ICUs between 1 January 2015 and 31 December 2019 inclusive, and for the RC alloantibody prevalence part of the study, who also had at least one blood group and antibody screen test performed by Territory Pathology at any time between 1 January 2015 and 31 December 2021. For analysis of new incident clinically significant RC alloantibodies, patients included were those with a RC antibody screen performed at or prior to index ICU admission (baseline) and ≥one RC antibody screen conducted after the ICU admission date. For time to event (alloimmunisation) analyses, patients were followed up to December 2021, with patients RC alloantibody-negative at the time of their last RC antibody screen test censured at that time point.

This study has ethics approval from the Human Research Ethics Committee of the Northern Territory Department of Health and Menzies School of Health Research, covering both involved institutions (approval reference NTHREC 2020–3930) and was conducted and reported according to the Strengthening the Reporting of Observational Studies in Epidemiology (STROBE) Statement [20].

### 2.2. Data Sources and Data Collection

Baseline demographics, including First Nations status, age at first index ICU admission during study period, sex, comorbidities, hospital episode, and ICU admission data (including diagnoses) were retrieved from the Australia and New Zealand Intensive Care Society Adult Patient Database (APD). The APD captures demographic, hospital, and ICU episode, clinical, physiological, and laboratory information of all patients admitted to ICUs across Australia and New Zealand [21]. Data are collected by trained staff who classify patients as Indigenous, non-Indigenous, or of unknown Indigenous status according to hospital and ICU medical records. Patients recorded as Indigenous on ANZICS APD database or Aboriginal and/or Torres Strait Islander on the NT Client Master Index were classified as First Nations. Where patient data on First Nations status were missing from both the ANZICS APD database and NT Client Master Index, the patient was excluded from the analysis.

ANZICS APD patient data were linked with blood transfusion data from the Territory Pathology laboratory information system, which provides transfusion laboratory services to all NT public hospitals, allowing linkage of patients’ NT-wide ABO and RhD blood groups, RC antibody screen and alloantibody identification/specificity, and total cumulative units of RCs transfused to patients from date of first ICU admission to the end of 2021. Linkage was performed by patient NT Hospital Record Number (HRN) and date of birth.

### 2.3. Alloantibody Screening and Identification

Antibody screening and identification were performed by an automated immunohaematology analyser (Ortho Vision, Ortho Clinical Diagnostics) and in accordance with Australia and New Zealand Society of Blood Transfusion guidelines [22]. In brief, all patient samples had an antibody screen performed, in which the patient’s plasma was tested by indirect antiglobulin test against a selected panel of three group O reagent RCs with known antigenic profiles. Patient samples with a positive antibody screen then proceeded to antibody identification using a panel of 11 group O reagent RCs, with the specificity of the single or multiple antibodies determined by comparing the pattern of reactions obtained against the reagent RC manufacturer’s cell panel antigen sheet. The antibody was then confirmed to be an alloantibody by testing to show that patient RC phenotype was antigen-negative for the identified antibody. Other supplementary techniques, for example, enzyme (e.g., Papain, which destroys Duffy and MNS RC antigens and enhances Kidd and Rhesus RC antigens), were also used to supplement the IAT technique where relevant.

### 2.4. Outcomes and Exposures of Interest

The primary outcome of interest for the analysis was the incidence of new clinically significant RC alloantibodies from time of index ICU admission during the study period. Clinically significant alloantibodies (usually of IgG type) were defined as per Table 1. To exclude any female patients with passively acquired anti-D RC alloantibodies because of anti-D immunoglobulin administration during pregnancy, all female patients with a RhD-positive alloantibody report who had a laboratory record of recent receipt of anti-D immunoglobulin during pregnancy and a laboratory issued report stating the alloantibody was passively acquired were not included as a RC alloantibody positive case. Secondary outcomes of interest included ABO and RhD blood groups, period prevalence of RC alloantibodies, and RC antibody specificity. Primary exposures of interest were sex, First Nations status, and cumulative RC transfusion exposure in units from time of index ICU admission date until 31 December 2021 or loss to follow up.

### 2.5. Statistical Analysis

Continuous variables were displayed as mean and standard deviation (SD), and categorical variables as frequencies and percentages. Comparisons for ABO and RhD blood groups between First Nations and non-First Nations patients and for independent predictor variables for new incident clinically significant RC alloantibody positive versus negative patients were performed by Pearson’s Chi^2^ (or Fisher’s exact test where indicated) for categorical independent variables, and for continuous variables, (Student’s) *t*-test for normally distributed data (or Kruskal–Wallis rank test for non-parametric data). Associations between the primary outcome of interest, new incident clinically significant RC alloantibody positivity, and potential predictor variables including age, sex, First Nations status, and cumulative RC transfusion exposure, were initially analysed by univariate Cox regression analyses and Kaplan–Meier curve analysis. Multivariate Cox proportional hazards regression models were then constructed using covariates of statistical significance on univariate analysis and those of biological or clinical importance (age, sex, First Nations status, RC transfusion exposure). The final model was selected according to the combination of potential predictors with the best fit. Estimate effects were reported as association hazard ratios (HR) and 95% confidence intervals (CI). A type 1 error of *p* < 0.05 was considered statistically significant. Statistical analysis was conducted by Stata for Windows, version 17.0, with the exception of graphs, which were generated in GraphPad Prism for Windows version 9.3.1.

**Table 1:** Red cell alloantibodies and their clinical significance. Derived from [23,24].

Blood Group System/Antigen		Clinical Significance	Immunoglobulin Type	Haemolytic Transfusion Reactions	Haemolytic Disease Fetus/Newborn	Clinical Significance Categorisation for This Study
002 MNS						
	M	Clinically insignificant if not reactive at 37 degrees	IgM or IgG	None	None to mild	No
	N	Clinically insignificant if not reactive at 37 degrees	IgM	None	None	No
	S	Usually clinically significant	IgG	None to moderate	None to severe	Yes
003 P1PK						
	P1	Not generally clinically significant	IgM	Rare	None	No
004 RH (Rhesus)						
	C	Usually clinically significant	IgG	Mild–severe	Mild	Yes
	D	Usually clinically significant	IgG or IgM	Mild–severe	Mild–severe	Yes
	E	Usually clinically significant	IgG or IgM	Mild–severe	Mild	Yes
	c	Usually clinically significant	IgG	Mild–severe	Mild–severe	Yes
	e	Usually clinically significant	IgG	Mild–moderate	Rare	Yes
	Cw	Usually clinically significant	IgG	Rare	Yes (in neonate occasionally)	Yes
005 LU (Lutheran)						
	Lu	Rarely clinically significant	IgG or IgM	None to mild	None	No
006 KEL (Kell)						
	K	Usually clinically significant	IgG or IgM	Mild–severe	Mild–severe	Yes
	Kp^a^	Usually clinically significant	IgG	Mild–moderate	Mild–moderate	Yes
007 LE (Lewis)						
	Le^a^	Clinically insignificant if not reactive at 37 degrees	IgM	Rare	None	No
	Le^b^	Not generally clinically significant	IgM	None	None	No
008 FY (Duffy)						
	Fy^a^	Usually clinically significant	IgG	Mild–severe	Mild–severe	Yes
	Fy^b^	Usually clinically significant	IgG	Mild–severe	Mild	Yes
009 JK (Kidd)						
	Jk^a^	Usually clinically significant	IgG	None–severe	Mild–moderate	Yes
	Jk^b^	Usually clinically significant	IgG	None–severe	None–mild	Yes
017 CH/RG (Chido/Rogers)						
	Rogers	Not generally clinically significant	Usually IgG	None	None	No

## 3. Results

**Figure 1:**
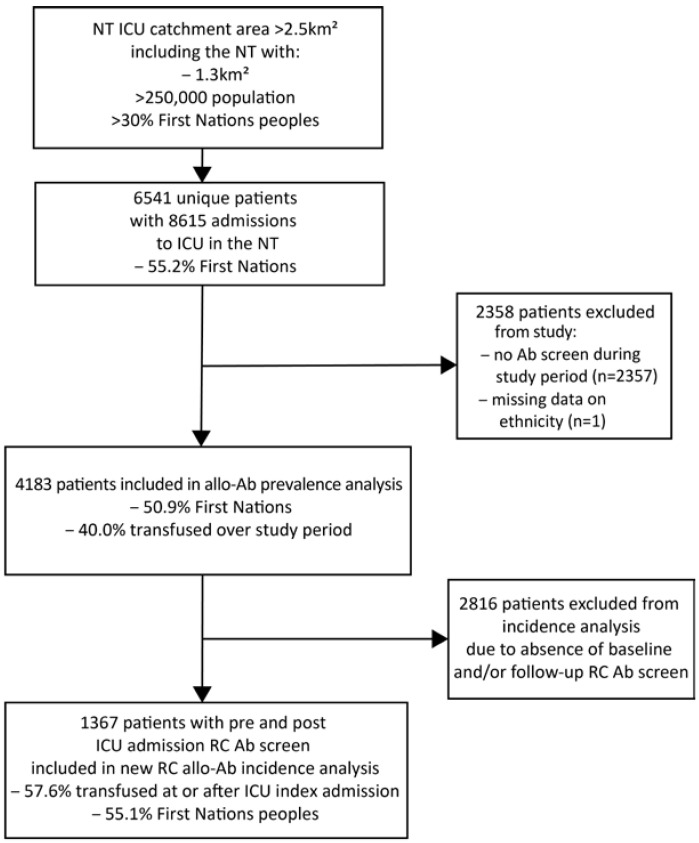
Study flow diagram, Northern Territory (NT) intensive care unit (ICU) patient admission retrospective cohort, 2015–2019 inclusive. Ab: antibody; RC: red cell.

From 2015–2019 inclusive, 6541 ICU unique patients were admitted to ICUs in the NT, with 8615 admissions in total (Figure 1). Of these, 2357 patients were excluded from the analysis due to not having had a blood group and RC antibody screen at any time during the study period, and one was excluded due to missing data on First Nations identification, leaving 4183 patients included in the study for the analysis for period prevalence and specificity of RC alloantibodies over the entire study period. In total, 1367 patients had a RC alloantibody screen performed both at baseline (prior to/on the day of admission to ICU) and on follow-up and were included in the study for the analysis of new incident RC alloantibodies.

### 3.1. Baseline Characteristics at Time of Index ICU Admission and Period Prevalence of RC Alloantibodies over the Entire Study Period

Of the 4183 total included patients, 50.9% were First Nations patients, and 45.9% were female. Mean (SD) age was 52.0 (17.7) years at time of index admission to ICU during the study period (Table 2). Of these 4183 patients, 68.3% were from the Top End region of the NT, and 31.8% from the Central region of the NT. Baseline characteristics were overall similar between the cohort of patients included in the prevalence part of the study (n = 4183) and new clinically significant RC alloantibody part of the study (n = 1367) (Table 2).

First Nations patients had significantly different ABO (*p* < 0.001) and RhD (*p* < 0.001) blood group distributions compared with non-First Nations patients (Table 3). First Nations patients were 55.9% group O compared with 46.5% of non-First Nations patients, and 98.8% RhD-positive compared with 84.5% of non-First Nations patients.

**Figure 2:**
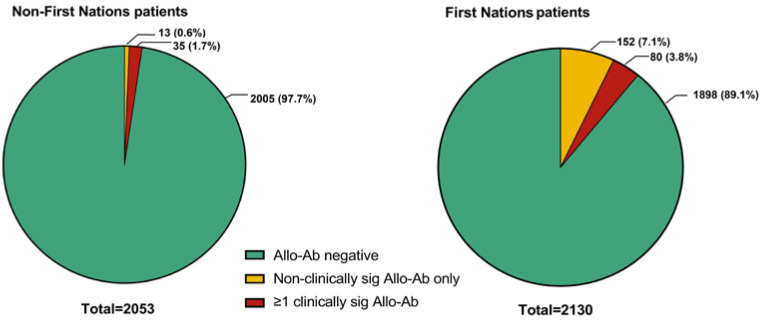
Period prevalence of patients with one or more clinically significant (sig) alloantibodies (Allo-Ab) versus non-clinically significant alloantibodies alone, grouped by First Nations status.

**Figure 3:**
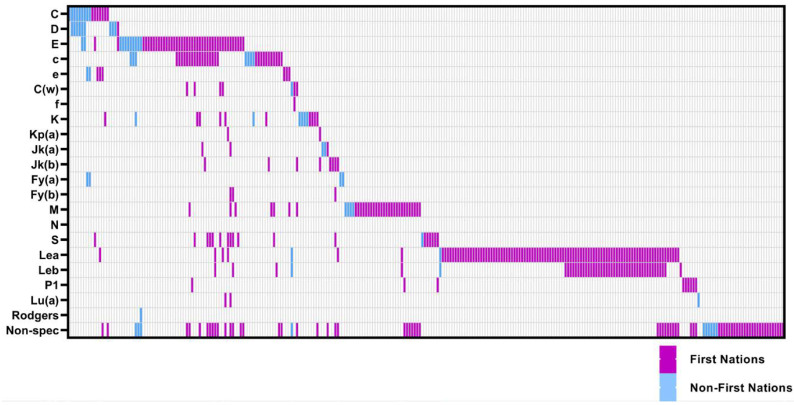
Distribution (specificity) of red cell alloantibodies in NT ICU patients (n = 280), First Nations (n = 232) versus non-First Nations (n = 48) patients. Each column represents an individual alloantibody-positive patient. Non-spec: non-specific agglutinin/antibody.

In total, 280 (6.7%) patients recorded a positive RC alloantibody screen across the entire study period. For First Nations patients across the entire study period, there were 390 prevalent alloantibodies detected in 232 total patients, compared with non-First Nations patients, with 72 alloantibodies detected in 48 total patients (Figure 2 and Figure 3, Table 4). Of the 232 RC alloantibody positive First Nations patients’ total 390 detected alloantibodies, the non-clinically significant types were relatively more prevalent: 188 (48.2%) were of non-clinically significant specificity (e.g., Le^a^, Le^b^, M, and P1), 135 (34.6%) were of clinically significant specificity, and 67 (17.2%) had no apparent identifiable specificity (Table 4). In contrast, of the 72 detected alloantibodies within the 48 non-First Nations alloantibody-positive patients, 10 (13.9%) were of non-clinically significant specificity, 52 (72.2%) were of clinically significant specificity, and 10 (13.9%) had no apparent identifiable specificity. However, when alloantibody prevalence as a proportion of all First Nations patients versus alloantibody prevalence as a proportion of all non-First Nations patients were compared, First Nations patients had higher absolute prevalence of both clinically significant (IgG type) and non-clinically significant (IgM type) alloantibodies overall compared with non-First Nations patients (Table 4): anti-E (positive in 2.0% of all First Nations patients versus 0.5% of all non-First Nations patients), anti-c (1.3% versus 0.3%), and Kell (0.5% versus 0.3%), respectively (Table 4).

### 3.2. Incidence and Predictors of New Clinically Significant RC Alloantibody Formation

For the 1367 patients with RC alloantibody testing performed at both baseline (at or prior to ICU index admission) and post-date of ICU admission, follow-up RC alloantibody screen testing was available at a median (IQR) of 69 (9–433) days post ICU admission day, equivalent to 1086 person years of follow-up. In these 1367 patients, new incident RC alloantibodies were detected in 70 patients, of which 62 were First Nations and 8 non-First Nations, equivalent to an incidence of 0.087 per person/year for First Nations versus 0.021 per person/year for non-First Nations patients. Of these 70 patients with new RC alloantibodies, 41 patients had clinically significant new incident RC alloantibodies (n = 34 First Nations patients and n = 7 non-First Nations patients, *p* < 0.001) (Table 5 and Table A1). Patients with new incident clinically significant RC alloantibodies received a median (IQR) of 6 (4–10) packed RC transfusions (units) during their follow up (from baseline index ICU admission date to end 2021 or loss to follow up), compared with a median (IQR) of 2 (0–5) RC transfusions (units) for patients who were negative for new incident clinically significant RC alloantibodies at follow up (*p* < 0.001). Patients with new incident clinically significant alloantibodies were significantly younger (mean (SD) 47.1 (14.7) years versus 53.1 (17.3), *p* = 0.01). Women were overrepresented in the new incident RC clinically significant alloantibody positive group (63%) compared with men (37%), but this difference was not statistically significant (*p* = 0.10).

**Figure 4:**
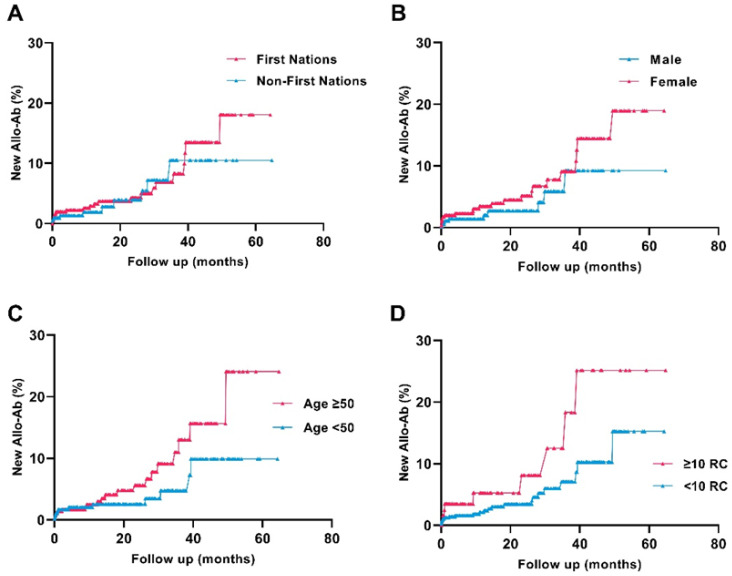
Kaplan–Meier curves for new incident clinically significant red cell alloantibodies (Allo-Ab) grouped by First Nations status (**A**), sex (**B**), age categorised as <50 or ≥50 (**C**), and cumulative red cell transfusion exposure from date of index ICU admission (categorised as <10 and ≥10 units packed red blood cells) (**D**).

On univariable analysis, hazard ratios (HR) for new incident clinically significant RC alloantibody positivity were significantly higher for First Nations patients (unadjusted HR 3.12 (95% CI 1.30–7.47)) and by cumulative exposure to RC transfusions over time (HR 1.02 (95% CI 1.01–1.05) per unit transfused) (Figure 4, Table 6). On Cox proportional hazards regression modelling, First Nations status (adjusted HR 2.67 (95% CI 1.05–6.80), *p* = 0.04) and cumulative RC transfusion exposure (adjusted HR per unit transfused 1.03 (95% CI 1.01–1.05), *p* = 0.01) were both independent predictors of RC alloimmunisation after adjustment for age and sex (Table 6).

**Table 6:** Alloantibody positivity 2015–2019: univariable and multivariable Cox proportional hazards regression of new incident clinically significant RC alloantibody-positive versus -negative patients admitted to ICUs in NT between 2015–2019.

	Unadjusted HR (95% CI)	*p*-Value	Adjusted HR (95% CI)	*p*-Value
Female sex	1.25 (0.64–2.42)	0.51	0.88 (0.43–1.77)	0.71
Age (years)	0.98 (0.96–1.00)	0.08	0.99 (0.96–1.01)	0.27
First Nations	3.12 (1.30–7.47)	0.01	2.67 (1.05–6.80)	0.04
RC units transfused post ICU admission (per unit)	1.02 (1.01–1.05)	0.01	1.03 (1.01–1.05)	0.01

HR: hazard ratio, CI: confidence interval; RC: red cells: ICU: intensive care unit.

## 4. Discussion

We present the first paper describing the prevalence, incidence, and predictors of RC alloantibodies in First Nations peoples of Northern Australia, using a five-year cohort of NT patients admitted to ICUs with critical illness. RC alloantibodies were present in significantly more First Nations patients compared with non-First Nations patients. First Nations status and RC transfusion exposure in units were both independently associated with a significantly increased hazard ratio for new clinically significant RC alloimmunisation. RC transfusion exposure was a statistically significant and clinically modifiable risk factor for RC alloimmunisation in our study.

The period prevalence of RC alloantibodies, including clinically significant ones associated with haemolytic transfusion reactions and haemolytic disease of the fetus/newborn (e.g., Rhesus, Kell, and Duffy), was higher in First Nations patients compared with non-First Nations patients. Our findings with respect to Kell (K) alloantibodies in First Nations patients are congruent with earlier smaller studies, including recent whole-genome sequencing studies on the distribution of blood group antigen profiles in First Nations Tiwi (n = 457) and Western Desert (n = 72) First Nations peoples [15,25]. Both these genomic studies found K antigen virtually absent in First Nations peoples compared with its presence in 9.1% (K) of Australian blood donors. In total, we report 10.9% of First Nations ICU patients being RC alloantibody-positive over the entire study period. This compares to a reported prevalence of 0.7% in blood donors [26] versus up to 18% in thalassaemia patients [27] and up to 47% in sickle cell disease patients [28]—both patient groups with chronic haematological disorders which confer a lifelong high RC transfusion requirement and amongst the highest reported rates of RC alloimmunisation of any population globally.

Our findings with respect to Rhesus alloantibodies in First Nations patients have significance for blood transfusion inventory management and highlight the potential risks in giving emergency issue group O RhD-negative blood, particularly in rural and remote settings. The high RhD-positive blood group prevalence in First Nations patients (nearly 99%) is consistent with previous reports on ABO and RhD blood groups in First Nations NT peoples. Our finding of c alloantibodies in 12% (28/232) of RC alloantibody-positive First Nations patients illustrates how the use of O RhD-negative blood for transfusion in emergencies or in remote settings where comprehensive transfusion services with ABO and RhD blood typing are not available—settings where many First Nations NT patients reside—can pose greater risk: in Caucasians, the most frequent Rhesus haplotype in RhD-negative blood donors is ce, compared with RhD-positive donors, which is Ce. Our findings imply that O RhD-positive RC for transfusion may have less Rhesus c antigen disparity for First Nations transfusion recipients [24,29]. This is illustrative of other broader evidence showing O RhD-negative emergency issued RC for transfusion are higher risk than group compatible or crossmatched RC for transfusion [30]. Our NT remote hospital laboratories serving First Nations communities now keep more emergency O RhD-positive inventory, with preference of giving O RhD-positive blood in emergency settings, helping to conserve Australia’s O RhD-negative blood supply [31].

Whilst overall absolute prevalence and incidence of clinically significant (IgG type) RC alloantibodies was higher in First Nations versus non-First Nations patients, within the subset of alloantibody positive First Nations patients, IgM type alloantibodies (e.g., Lewis and M) were relatively more common. The majority of these are typically clinically insignificant; however, in First Nations peoples in rural and remote settings, this may not be so [32]. Such alloantibodies can prove challenging with respect to finding compatible RCs for future transfusions in a timely fashion, leading to delays in medical care [8]. It is unclear why the relative prevalence of IgM alloantibodies in First Nations patients was much higher that the IgG type, and the reverse being true for non-First Nations patients. Of note, IgM antibodies can be naturally occurring and induced by non-RC transfusion or pregnancy-related host factors [8]. In addition, whilst donor–recipient discrepancies among RC antigens is necessary for RC alloimmunisation, only a fraction of patients exposed to such antigens develop RC alloantibodies. Additional transfusion recipient factors, such as HLA type and antigen recognition ability, and concurrent autoimmune or inflammatory states also play a role in increasing the risk of RC alloimmunisation [8]. Further studies are recommended to explore any association with these other factors and risk of RC alloimmunisation in First Nations peoples, as well as the implications of RC alloantibodies (including those traditionally considered non-clinically significant) on transfusion logistics, especially in remote healthcare settings.

Whilst our study has many strengths, there are also some limitations, including its retrospective design and reliance on routinely collected clinical and laboratory data. Loss to long-term follow-up is typical of both retrospective and prospective cohort studies and particularly important in research on RC alloimmunisation, as RC alloantibodies have a known incidence of evanescence and anamnestic response over time [8]. The kinetics of such RC alloantibody persistence versus evanescence remain unknown for First Nations patients. Moreover, given the likelihood that patients with higher re-hospitalisations, comorbidities, and future RC transfusions are more likely to undergo long-term repeated blood group and antibody screens, it is likely that our data have a selective inclusion of patients at higher risk of long-term RC alloantibody development. The strengths of our study include its 100% coverage of all ICU admissions across the NT, the availability of a single unique hospital record number patient identifier across all NT hospitals and laboratories, allowing laboratory and clinical data linkage across all NT sites, and a single NT-wide transfusion service provider, enabling accurate linkage of longitudinal RC transfusion exposure. Given the size of our cohort and the statistical significance of our findings, our study provides the best evidence, to date, on RC alloimmunisation risk in First Nations Australians.

Our findings underscore the importance of very judicious use of RC transfusions, particularly in the context of international studies that demonstrate the safety of lower, more restrictive triggers of haemoglobin for RC transfusion [33]. Consideration of alternative treatment strategies to avoid transfusion where possible are paramount. The increased risk of alloimmunisation in First Nations peoples should be considered in the assessment of risks and benefits of transfusion. Our findings highlight the importance of decolonising approaches to decision-making in RC transfusion: this must include genuine understanding of risks and benefits by prescribers of transfusions, informed consent, and true shared decision-making with patients within a culturally and linguistically supported environment, framed by training for clinicians regarding the special cultural and spiritual significance that blood holds for First Nations peoples [14,34,35,36]. Future prospective studies are recommended to further clarify other RC recipient factors associated with the development of RC alloantibodies in First Nations patients, the consequences of them, including those traditionally considered as non-significant, and their kinetics with respect to alloantibody persistence or evanescence. Such studies should include clinical and laboratory outcome measures which will help establish the significance of these alloantibodies, such as incidence of haemolytic transfusion reactions and haemolytic disease of the newborn/fetus, and time to finding compatible RCs for transfusion in rural and remote contexts. Genomics research also offers immense capacity for understanding the distribution and variants in RC antigens in different populations, although genetic research in First Nations peoples requires sound approaches with respect to community engagement, co-design, First Nations leadership, and governance [37].

## Figures and Tables

**Table 2:** Baseline characteristics at time of index ICU admission for all patients admitted to ICU with at least one RC antibody screen performed over the study period (2015–2019).

			All Patients Included in Prevalence Cohort of Study	Patients Included in Incidence Cohort Part of Study
Patients, n			4183	1367
Sex, n (%)				
	Male		2265 (54.2%)	672 (49.2%)
	Female		1918 (45.9%)	695 (50.8%)
First Nations, n (%)				
	First Nations		2130 (50.9%)	753 (55.1%)
	Non-First Nations		2053 (49.1%)	614 (44.9%)
Region/district, n (%)				
	Top End		2855 (68.3%)	963 (70.5%)
	Central		1328 (31.8%)	404 (29.6%)
Age at index ICU admission, years, mean SD			52.0 (17.7)	52.9 (17.3)
Pregnancy status at index ICU admission n, (% females)				
	Not pregnant		1726 (90.0%)	622 (89.5%)
	Currently pregnant		55 (2.9%)	30 (4.3%)
	Post-partum		137 (7.1%)	43 (6.2%)
Index admission diagnosis, n (%)				
	Sepsis		576 (13.8%)	192 (14.0%)
	Trauma		756 (18.1%)	211 (15.4%)
	Gastrointestinal		610 (14.6%)	282 (20.6%)
	Cardiovascular		555 (13.3%)	171 (12.5%)
	Respiratory		539 (12.9%)	109 (8.0%)
	Neurological		371 (8.9%)	83 (6.1%)
Comorbidities, n (%)				
	Immune disease		49 (1.2%)	24 (1.8%)
	Immunosuppressed		131 (3.1%)	56 (4.1%)
	Diabetes mellitus			
		Type 1 DM	15 (0.4%)	6 (0.4%)
		Type 2 DM	465 (11.2%)	154 (11.3%)
		Gestational diabetes	15 (0.4%)	5 (0.4%)
	Chronic renal disease		531 (12.7%)	249 (18.2%)
	Hepatic failure		35 (0.84%)	11 (0.8%)
	Cirrhosis		167 (4.0%)	86 (6.3%)
	Chronic respiratory disease		236 (5.6%)	61 (4.5%)
	Chronic cardiovascular disease		288 (6.9%)	108 (7.9%)

ICU: intensive care unit; SD: standard deviation; DM: diabetes mellitus.

**Table 3:** ABO and RhD blood groups in First Nations versus non-First Nations patients.

ABO RhD Blood Group		Total (n = 4183)	First Nations (n = 2130)	Non-First Nations (n = 2053)	*p*-Value
ABO					<0.001
	O	2145 (51.3%)	1191 (55.9%)	954 (46.5%)	
	A	1638 (39.2%)	876 (41.1%)	762 (37.1%)	
	B	310 (7.2%)	52 (2.4%)	258 (12.6%)	
	AB	90 (2.2%)	11 (0.5%)	79 (3.8%)	
RhD					<0.001
	RhD +	3839 (91.8%)	2104 (98.8%)	1735 (84.5%)	
	RhD −	344 (8.2%)	26 (1.2%)	318 (15.5%)	

RhD: Rhesus D blood group; RhD +: RhD antigen positive patient; RhD −: RhD antigen negative patient.

**Table 4:** Specificities of prevalent RC alloantibodies across entire study period by First Nations status.

RC Blood Group System/Antigen Specificity of RC Allo-Ab		Total n	First Nations Patients			Non-First Nations Patients		
			n	% of +ve Allo-Ab Patients (n = 232)	% of All Patients (n = 2130)	n	% of +ve Allo-Ab Patients (n = 48)	% of All Patients (n = 2053)
**Total patients with RC allo-Ab, n**		280	232	-	10.9%	48	-	2.3%
**Total RC allo-Ab, n**		462	390	-	-	72	-	-
**002 MNS**								
	M	37	33	14.2%	1.6%	4	8.3%	0.2%
	N	0	0	0.0%	0.0%	0	0.0%	0.0%
	S	19	18	7.8%	0.8%	1	2.1%	0.0%
**003 P1PK**								
	P1	9	9	3.9%	0.4%	0	0.0%	0.0%
**004 RH (Rhesus)**								
	C	16	7	3.0%	0.3%	9	18.8%	0.4%
	D	10	1	0.4%	0.0%	9	18.8%	0.4%
	E	53	42	18.1%	2.0%	11	19.0%	0.5%
	c	35	28	12.1%	1.3%	7	14.6%	0.3%
	e	8	6	2.6%	0.3%	2	4.2%	0.1%
	f	1	1	0.4%	0.0%	0	0.0%	0.0%
	Cw	7	6	2.6%	0.3%	1	2.1%	0.0%
**005 LU (Lutheran)**								
	Lu(a)	3	2	0.9%	0.1%	1	2.1%	0.0%
**006 KEL (Kell)**								
	K	16	10	4.3%	0.5%	6	12.5%	0.3%
	Kp^a^	2	2	0.9%	0.1%	0	0.0%	0.0%
**007 LE (Lewis)**								
	Le^a^	101	99	42.7%	4.6%	2	4.2%	0.1%
	Le^b^	47	45	19.4%	2.1%	2	4.2%	0.1%
**008 FY (Duffy)**								
	Fy^a^	4	0	0.0%	0.0%	4	8.3%	0.2%
	Fy^b^	3	3	1.3%	0.1%	0	0.0%	0.0%
**009 JK (Kidd)**								
	Jk^a^	5	3	1.3%	0.1%	2	4.2%	0.1%
	Jk^b^	8	8	3.4%	0.4%	0	0.0%	0.0%
**017 CH/RG (Chido/Rodgers)**								
	Rg1	1	0	0.0%	0.0%	1	2.1%	0.0%
**No apparent specificity**		77	67	28.9%	3.1%	10	20.1%	0.5%

Allo-Ab: alloantibody; RC: red cell.

**Table 5:** Characteristics of patients at time of index ICU admission in NT public hospitals during the study period (2015–2019), for patients with both a baseline and follow-up RC alloantibody screen performed, categorised by new incident clinically significant RC alloantibody status.

		Total	Clinically Significant Alloantibody-Positive	Clinically Significant Alloantibody-Negative	*p*-Value *
N (% total) unique patients		1367	41	1326	
Sex					0.10
	Male, n (%)	672 (49.2%)	15 (37%)	657 (50%)	
	Female, n (%)	695 (50.8%)	26 (63%)	669 (51%)	
First Nations					<0.001
	First Nations, n (%)	753 (55.1%)	34 (83%)	719 (54%)	
	Non-First Nations, n (%)	614 (44.9%)	7 (17%)	607 (46%)	
Region/district					
	Top End, n (%)	963 (70.5%)	29 (71%)	934 (70%)	
	Central, n (%)	404 (29.6%)	12 (29%)	392 (30%)	
Age at index ICU admission (years), mean (SD)		52.9 (17.3)	47.1 (14.7)	53.1 (17.3)	0.01
Haemoglobin nadir g/L, during Day 1 ICU first admission, mean (SD)		91 (23)	74 (15)	92 (23)	<0.001
Cumulative number of red cell unit transfusions from baseline index ICU admission date to end 2021 per patient (median, IQR)		2 (0–5)	6 (4–10)	2 (0–5)	<0.001 **
Pregnancy status at time of index ICU admission (% females)					0.21
	Not pregnant	622 (89.5%)	26 (100%)	596 (89%)	
	Currently pregnant	30 (4.3%)	0 (0%)	30 (4%)	
	Post-partum	43 (6.2%)	0 (0%)	43 (6%)	
Comorbidities					
	Immune disease	24 (1.8%)	1 (2%)	23 (2%)	0.74
	Immunosuppressed	56 (4.1%)	1 (2%)	55 (4%)	0.59
	Chronic renal disease	249 (18.2%)	10 (24%)	239 (18%)	0.30
	Hepatic failure	11 (0.8%)	1 (2%)	10 (1%)	0.23
	Chronic respiratory	61 (4.5%)	2 (5%)	59 (5%)	-
	Chronic cardiovascular	108 (7.9%)	2 (5%)	106 (8%)	0.47

IQR: interquartile range; * Student’s *t*-test for difference between means for continuous variables, Chi^2^ or Fisher’s exact for categorical variables; ** Kruskal–Wallis rank test.

## Data Availability

Data used for this study are not publicly available due to ethical, privacy, and sensitivity considerations. Data may be available from the corresponding author on reasonable request and subject to relevant ethical approvals.

## Appendix A

**Table A1:** Specificities of new incidence RC alloantibodies across entire study period by First Nations status.

Red Cell Blood Group System/Antigen Specificity of Allo-Ab		Total n	First Nations Patients			Non-First Nations Patients		
			n	% of +ve Allo-Ab Patients (n = 62)	% of All Patients (n = 753)	n	% of +ve Allo-Ab Patients (n = 8)	% of All Patients (n = 614)
**Total patients with new RC allo-Ab, n**		70	62	-	8.2%	8	-	1.3%
**Total RC allo-Ab, n**		121	106	-	-	15	-	-
**002 MNS**								
	M	5	5	8%	0.7%	0	0%	0%
	N	0	0	0%	0.0%	0	0%	0.0%
	S	7	7	11%	0.9%	0	0%	0.0%
**003 P1PK**								
	P1	4	4	6.5%	0.5%	0	0.0%	0.0%
**004 RH (Rhesus)**								
	C	3	2	3%	0.3%	1	13%	0.2%
	D	1	1	2%	0.1%	0	0%	0.0%
	E	22	18	29%	2.4%	4	50%	0.7%
	c	12	10	16%	1.3%	2	25%	0.3%
	e	4	3	5%	0.4%	1	13%	0.2%
	f	0	0	0%	0.0%	0	0%	0.0%
	Cw	3	3	5%	0.4%	0	0%	0.0%
**005 LU (Lutheran)**								
	Lu(a)	2	2	3%	0.3%	0	0%	0.0%
**006 KEL (Kell)**								
	K	5	4	6%	0.5%	1	13%	0.2%
	Kp^a^	1	1	2%	0.1%	0	0.0%	0.0%
**007 LE (Lewis)**								
	Le^a^	6	6	10%	0.8%	0	0.0%	0.0%
	Le^b^	6	6	10%	0.8%	0	0.0%	0.0%
**008 FY (Duffy)**								
	Fy^a^	1	0	0.0%	0.0%	1	13%	0.2%
	Fy^b^	2	2	3%	0.3%	0	0.0%	0.0%
**009 JK (Kidd)**								
	Jk^a^	3	1	2%	0.1%	2	25%	0.3%
	Jk^b^	5	5	8%	0.7%	0	0.0%	0.0%
**017 CH/RG (Chido/Rodgers)**								
	Rg1	1	0	0.0%	0.0%	1	13%	0.2%
**No apparent specificity**		28	26	41.9%	3.5%	2	25%	0.3%
